# Biofilms: Their Role in Dermal Fillers

**DOI:** 10.4103/0974-2077.63257

**Published:** 2010

**Authors:** Anitha B Sadashivaiah, Venkataram Mysore

**Affiliations:** *Venkat Charmalaya, Centre for Advanced Dermatology, Bangalore, Karnataka, India*

**Keywords:** Aesthetics, biofilm, fillers

## Abstract

Fillers are commonly used in several aesthetic indications. Though considered safe, several side effects have been reported. The role of biofilms in the causation of some of these side effects has been elucidated only recently and this article presents a short review of the subject.

## INTRODUCTION

The concept of biofilm is relatively new to dermatology, though it is a well-established concept in other fields. Recently, it has gained importance as it is often encountered in patients who have received long-term filler implants for aesthetic improvement. This article outlines this concept and its relevance to dermatosurgeons.

## DEFINITION

A biofilm is an aggregate of microorganisms in which cells are stuck to each other and/or to a surface, embedded within a self-secreted extracellular protective and adhesive matrix of a polymeric substance (EPS).[1] Biofilms are usually found on solid substrates submerged in or exposed to some aqueous solution. They are widely distributed in nature. Biofilms can form in different situations in the human body, such as attachment on a surface, or after exposure to sublethal doses of antibiotics.[2] Such surfaces may be provided by artificial devices such as prosthetic valves and breast implants or in dermatological situations like with fillers, and cheek implants. A biofilm may also form around an infective focus, particularly when a patient is administered inadequate doses of antibiotics.

## THE PROCESS OF BIOFILM FORMATION

While biofilms can contain many different types of microorganisms, e.g., bacteria, protozoa and fungi, a biofilm model colonized by *Pseudomonas aeruginosa* has been studied extensively. The various stages during the development of a biofilm have been elucidated and they include,[2]

- attachment of bacteria to the surface
- microcolony formation
- biofilm maturation
- dispersion

The formation of a biofilm begins with the localization, concentration and attachment of free-floating bacteria around a surface which is usually in and around an infective focus. The bacteria get trapped along with cells (leucocytes) in the EPS, forming microcolonies, which makes them unsusceptible for being attacked by the antibiotics. EPS is composed of DNA, proteins and polysaccharides. This matrix protects the cells within it and also facilitates communication among them through biochemical signals. These chemical signals facilitate the distribution of nutrients to the growing bacteria in the biofilm.[2] The bacterium then enters into the biofilm growth phase, and undergoes a phenotypic shift in behaviour in which large suites of genes are differentially regulated.[3] The biofilm grows through a combination of cell division and recruitment. The final stage of biofilm formation is known as the development phase, in which the biofilm is fully established and may only change in shape and size. Such fully developed bacterial colony(ies) tends to be antibiotic resistant. The bacteria also acquire flagella, pili, DNA and EPS, during these different stages of their development. They also secrete bioactive substances which are not produced by the non-aggregated bacteria of the same species. The dispersion of the biofilm may lead to the spread and formation of new colonies.

The biofilm can either be dormant or active depending upon the external triggering factor. When the cell metabolism shuts down, it becomes dormant (persister). Thus it gets out of reach for antibiotics and also becomes difficult to culture *in vitro*. It becomes active following any disturbance in its local environment, such as trauma, injection, manipulation, resulting in manifestations such as local low-grade infection, abscess, local lumps, foreign body granuloma, nodule or systemic infection.[4]

## CLINICAL SIGNIFICANCE OF BIOFILMS

The importance of the biofilms to the clinician lies in the fact that they have been implicated in several common infectious processes, such as urinary tract infections, catheter infections, middle-ear infections, formation of dental plaque, gingivitis,[5] coating contact lenses[6] and intrauterine devices.[7] Serious and lethal processes such as endocarditis, infections in cystic fibrosis and infections of permanent indwelling devices such as joint prostheses and heart valves may also be associated with biofilms.[2,8] Since they are resistant to bacteria, they tend to persist and provide a focus for further activation and spread of infection.

### Biofilms in dermatosurgery

The importance of biofilms to the cutaneous surgeon has been realized only recently. It has been shown that bacterial biofilms may impair cutaneous wound healing and reduce topical antibacterial efficiency in healing or treating infected skin wounds.[9]

The role of biofilms in filler-induced adverse reactions has received increasing attention. Many adverse reactions have been reported after the administration of fillers, such as nodules, abscesses, sinuses, delayed reactions, etc. Such reactions, though uncommon, may occur, particularly with long acting fillers. They develop within weeks after the administration of the filler, and present as erythematous, mildly tender nodules. They often persist for months and cause great anxiety to the patient. They are usually culture negative and hence they were previously thought to be due to an allergic or a foreign body reaction to the filler substance. However, supporting data for such an allergic hypothesis have been lacking. These reactions are always small, localized and have no associated antibody formation. Further, many of them resolve with the use of antibiotics. These reactions, particularly those occuring after administration of hydrophillic fillers are now thought to be the consequence of biofilms.[4] Further proof of their infective aetiology has been provided by a recent article which showed that fluorescence *in situ* hybridization could demonstrate bacteria in seven out of eight biopsies, which were culture negative. Such techniques therefore could be of greater benefit in establishing the infectious cause of such nodules.[10,11]

The recognition of the concept of biofilm as the cause of such nodules has great relevance for their management. Hitherto, they were managed by the administration of corticosteroids, either intralesional or systemic.[4] For obvious reasons, steroids may worsen the condition. Diagnosis of any tender nodule over any implant should therefore be treated promptly by the administration of broad-spectrum bactericidal antimicrobials for 2–3 weeks. If any steroid injection is planned, it should be done only after the administration of a course of antibiotics.

## PREVENTION OF BIOFILMS

The prevention of biofilms is of great importance in this commonly performed aesthetic procedure. Filler injections are performed in an office setting and often in areas with high numbers of resident bacteria such as lips, and facial skin with acne. Therefore, proper standard of care should be adopted to prevent any infection by proper pre- and post-procedure aseptic precautions.

- Thorough cleansing of the area by an antiseptic solution such as povidone iodine before the filler injection is necessary.
- Local application of mupirocin after injection of fillers is advocated.
- The patient should be informed to report any tenderness developing after injection in the treated area, which should be promptly treated with antibiotics.
- Prophylactic antimicrobial therapy, with a single dose of a broad-spectrum antibiotic in the prevention of biofilm, has been recommended, but its role is not fully established.

## CONCLUSION

It is important for the aesthetic dermatologist to understand the role of infection and biofilms as a complication of filler injections. Further studies and research are therefore essential to unfold the mystery around biofilms.
